# Neural Correlates of True and False Memory in Mild Cognitive Impairment

**DOI:** 10.1371/journal.pone.0048357

**Published:** 2012-10-31

**Authors:** Catherine M. Sweeney-Reed, Patricia M. Riddell, Judi A. Ellis, Jayne E. Freeman, Slawomir J. Nasuto

**Affiliations:** 1 Memory and Consciousness Research Group, University Clinic for Neurology and Stereotactic Neurosurgery, Medical Faculty, Otto von Guericke University, Magdeburg, Germany; 2 School of Psychology and Clinical Language Sciences, University of Reading, Earley Gate, Reading, United Kingdom; 3 Cybernetics Research Group, School of Systems Engineering, University of Reading, Whiteknights, Reading, Berkshire, United Kingdom; Tohoku University, Institute for Development, Aging and Cancer, Japan

## Abstract

The goal of this research was to investigate the changes in neural processing in mild cognitive impairment. We measured phase synchrony, amplitudes, and event-related potentials in veridical and false memory to determine whether these differed in participants with mild cognitive impairment compared with typical, age-matched controls. Empirical mode decomposition phase locking analysis was used to assess synchrony, which is the first time this analysis technique has been applied in a complex cognitive task such as memory processing. The technique allowed assessment of changes in frontal and parietal cortex connectivity over time during a memory task, without a priori selection of frequency ranges, which has been shown previously to influence synchrony detection. Phase synchrony differed significantly in its timing and degree between participant groups in the theta and alpha frequency ranges. Timing differences suggested greater dependence on gist memory in the presence of mild cognitive impairment. The group with mild cognitive impairment had significantly more frontal theta phase locking than the controls in the absence of a significant behavioural difference in the task, providing new evidence for compensatory processing in the former group. Both groups showed greater frontal phase locking during false than true memory, suggesting increased searching when no actual memory trace was found. Significant inter-group differences in frontal alpha phase locking provided support for a role for lower and upper alpha oscillations in memory processing. Finally, fronto-parietal interaction was significantly reduced in the group with mild cognitive impairment, supporting the notion that mild cognitive impairment could represent an early stage in Alzheimer’s disease, which has been described as a ‘disconnection syndrome’.

## Introduction

Alzheimer’s disease (AD) is a neurodegenerative process responsible for fifty to sixty percent of cases of dementia over the age of 65 [1]. Early recognition of prodromal signs is increasingly important as advances are made in treatment, so that preventative or symptom-delaying medications may be started by arrest of the pathological processes before irreversible damage occurs [2], [3], [4], [5]. Furthermore, better prediction of progression to AD is essential for planning patient care. The well-recognised neuropathological changes found in AD are also frequently found in mild cognitive impairment (MCI), and therefore it is possible that MCI represents an early stage of the same process [6], [7]. MCI can be defined as subjective memory impairment and reduced memory for age, but typical general cognitive function and activities of daily living and no dementia, and using this definition, 12% of patients with MCI develop AD per year [8]. Detection of underlying neural mechanisms that differ between those with normal memory and those with MCI should lead to refinement of future diagnostic tests, allowing more accurate prediction of which people with MCI will go on to develop AD, and of this group, who will benefit from specific treatment approaches. Furthermore, a greater understanding of the electrophysiological correlates of memory processing may in future allow development of new treatment approaches, including both pharmacological and electrical. Our aim was to explore differences in the neural correlates of true and false memory to characterise how memory processing differs between typical older people and those with MCI.

The electroencephalogram (EEG) provides a potential low-cost, non-invasive method to improve early diagnosis as well as a means to enhance our understanding of the cortical processes affected. Here, we primarily investigated the use of phase synchrony in EEG recorded from participants with MCI and age-matched controls during true and false memory, calculated using a recently introduced analysis technique, empirical mode decomposition phase locking (EMDPL) [9]. The method has been introduced and compared with other approaches to phase synchrony analysis elsewhere [9], [10], but as we introduce the method here for the first time to the study of memory, we also provide a comparison with traditional bandpass filtering and wavelet based approaches, both to illustrate the advantages offered by EMDPL, as well as to validate our findings. Amplitude information was also determined following empirical mode decomposition and wavelet time-frequency decomposition to investigate whether amplitude differences could provide complementary information to highlight inter-group differences. Finally, event-related potentials (ERPs) were measured, to provide a benchmark comparison with previous ERP studies investigating true and false memory processing in such groups. Here, we will introduce the reasoning behind our choice of approach, before providing a brief review of what is already known about the behavioural and neurophysiological correlates of true and false memory.

Phase synchrony was chosen for this study as it is thought to be of fundamental importance in cognitive processing [11], [12] and to be affected in AD [2], [13], making it a potentially useful measurement to detect differences in neural processing between participants with MCI and age-matched controls. It has been postulated that synchronisation is the mechanism by which information processed by functionally separate brain regions is integrated for cognitive functions, including memory processing [11], [12]. The transient, dynamic neural assemblies resulting from activation of different brain regions over time, which are mediated by synchrony in particular frequency ranges, can be studied in electrophysiological data such as the EEG [11], [14], [15].

Short and long range synchrony, that is synchronous EEG activity between adjacent and distant brain areas respectively, have already been identified during memory tasks in typical participants and those with memory impairment [15], [16], [17], [18], [19], [20], [21], [22]. The findings indicate that synchrony can be useful in separating these participant groups both using resting EEG [13], [16], [23], [24], [25], [26], [27], [28] and EEG recorded during a relevant task [29], [30], [31], [32]. However, differences between typical and MCI groups are not always detected at rest. For example, in a study of EEG power differences between normal and MCI groups, differences were detected during working memory tasks but not at rest [33]. For this reason, we chose to record EEG during a memory task.

Performance has been shown to differ between typical participants and those with MCI in tasks known to increase the likelihood of false memory, and therefore we chose to measure EEG correlates of the Deese-Roediger-MacDermott (DRM) paradigm. In addition to group differences in this task, true and false memories have been demonstrated to show different neural signatures, at least in typical young participants [34]. EEG measured during this task therefore has the potential to shed light on how true versus false memory processing changes in patients with MCI, as well as to differentiate between participant groups. As far as we are aware, this is the first study in which phase synchrony has been used to explore false memory in patients with MCI.

### 1. Paradigm

False memory was introduced by [35] and demonstrated in simple list learning tasks by [36], where participants who had viewed a list of related words would falsely recall the presence of a word associated with the list, often termed the lure word. This task was extended to include a recognition memory task [37], and used by [38] with extended word lists to show that typical participants falsely recalled, and confidently recognised, the lure in 40% of trials. The paradigm used in the current study was a version of the task used by [38], known as the DRM paradigm.

### 2. Phase Synchrony

An ideal measure of the activity of distributed neural networks would allow measurement of changes in the synchronous firing patterns within the network over time. Typically, synchrony has been calculated using coherence, a measure reflecting both amplitude and timing information. Furthermore, the dynamic changes in synchrony over time are often not considered. It has been suggested that phase or timing information alone, independent of amplitude information, provides a mechanism by which functionally separate regions of the brain interact [11]. In this study, we therefore calculated instantaneous phase using EMDPL, allowing us to investigate changes in connectivity over time.

Phase locking (PL) must be calculated in a narrow frequency band for the phase to be interpretable, which usually necessitates a priori selection of frequency range cut-offs. Previous EEG studies have indicated that particular frequency ranges are associated with different aspects of cognition, but there is little consistency in the precise choice of the frequencies included in a given range. The precise corner frequencies chosen to define the particular frequency bands have an important influence on phase synchrony detection. Firstly, the frequency range relevant to a particular task varies between participants [10], [39], [40], [41], [42] and secondly, the cut-offs can influence whether phase synchrony is even detected [9], [10], [43]. We introduced EMDPL to the study of phase synchrony in EEG signals in order to address this problem [9], [10], [44], [45]. The method is an extension of EMD, an approach to time-frequency analysis of broadband signals initially developed by [46] for the analysis of geophysical data, which has since been utilised in a broad range of applications, from engineering and biomedical to financial time series analysis [46], [47], [48], [49], [50], [51], [52].

EMD is an iterative sifting process by which a broadband signal is decomposed into frequency modulated components whose definition allows calculation of instantaneous amplitude, phase, and frequency using the Hilbert transform (HT) [46]. We first explored the use of EMD to isolate neural assemblies mediated by brief episodes of phase synchrony [44], [45], and subsequently extended the method to introduce EMDPL analysis, which incorporates a statistical framework for the assessment of the significance of episodes of PL and also the EMDPL spectrum [9]. The latter is a phase synchrony adaptation of the Hilbert spectrum (HS), in which episodes of significant synchrony between a pair of signals are plotted against instantaneous frequency and time. Both application to artificial signals, and to real EEG data in the context of a simple motor task, demonstrated that EMDPL permits successful time-frequency localisation of PL between broadband signals [10], [53]. In the current study, the assessment of statistical significance was extended to permit comparison between different study conditions.

### 3. Frequencies

In previous studies, theta oscillations have been shown to be involved in episodic memory, and upper alpha oscillations have been associated with long-term memory in particular [15], [54]. Lower alpha oscillations have been postulated to play a role in attention [55], which may also be relevant, as attentional deficits are thought to contribute in AD [56], [57]. On the basis of this literature we chose oscillations in the theta and alpha ranges as the focus of the study. We postulated that synchronisation changes related to memory and to attention would be detected in patients with MCI before the development of AD, demonstrated by differences between people with MCI compared to typical older adults.

While review of the literature indicates that theta and alpha frequency ranges are likely to be relevant to the task, the cut-offs for these ranges differ greatly between studies. For example, the theta range has been defined as 3–5 Hz [58], 3.9–6.8 Hz [21], 4–6 Hz [59], 4–7 Hz [19], 4–7.5 Hz [31], 4–8 Hz [16], and 4.5–7.5 Hz [13]. Similar variation in alpha frequency ranges have also been reported [10], [39], [54]. Since EMDPL is data-driven on a subject-by-subject, trial-by-trial basis and does not require a priori selection of frequency ranges for analysis, relevant information is not lost by a priori parameter selection [10].

### 4. Brain Regions

Frontal and parietal areas have been shown to be important in memory processing using a variety of neuroimaging techniques [19], [21], [34], [60], [61], [62], [63], [64], [65], [66], [67]. More specifically, the right frontal and left parietal regions were selected for study here, both on the basis of evidence for their involvement in recognition memory [65], [68], [69], [70], [71], [72], [73], and also following neuropsychological and lesion studies, which have identified neural correlates of true versus false memory in these areas (reviewed by [74]). Indeed, true and false recognition have elicited prefrontal and parietal cortex as well as hippocampal activity [75], [76], all areas commonly involved in episodic memory retrieval [74].

False memory has been demonstrated to change with age and in disease, occurring more readily with increasing age [77], in MCI and AD [78], [79], [80], and in frontal lobe damage [81], [82], [83]. The increase in false recognition found in patients with frontal damage has led to the suggestion that frontal areas have a role in reducing or avoiding false episodic memory [74]. This provides a potential mechanism for the reduction in suppression of false memories in patients with AD [84] since frontal regions have been found to be affected by AD [84], [85], [86], [87], [88]. One hypothesis is that impaired frontal lobe function could result in increases in false memory via a combination of semantic memory network breakdown alongside reduced attentional control and inhibition. It has been suggested, for instance, that participants with frontal lobe damage might rely excessively on similarities between the items presented [74] as a result of monitoring deficits [74], [83], [89], [90]. Furthermore, investigation of recognition memory has revealed dissociations that have given rise to a number of dual-process models, in which recognition is taken to involve either recollection of past events or assessment of familiarity [91]. At least partially separate neural networks are thought to underlie these two processes, with recollection involving the hippocampus, anterior thalamus, and pre-frontal cortex, and familiarity requiring the parahippocampal gyrus, the dorsomedial thalamus, and the frontal lobes [92]. In particular, the two processes are believed to be separate at retrieval, and indeed, ERPs differ between the two. Thus, differences in ability to recognise past events between groups might be expected to result from differences in activation of the familiarity network, which includes the frontal lobe. Patients with MCI are hypothesised to have a greater tendency to rely on familiarity, since this has been shown to be spared relative to recollection [93]. On the basis of these findings, we postulated that reliance on different types of memory processing would result in differences in frontal neural processing underlying false memory between participant groups and therefore that this would provide one way to differentiate between the groups in the absence of a difference in performance.

Evidence also suggests a contribution of parietal lobe activation during memory tasks [66]. For instance, left parietal theta power has been shown to increase during retrieval [71]. It has been suggested that parietal evoked activity (power increase) and PL may indicate simultaneous activation of storage networks [94]. Furthermore, central executive processes, reflecting attention and the interface between working and long-term memory [19], [95], [96], have been postulated to result from functional connectivity between frontal and parietal areas forming a fronto-parietal network [16], [21]. [19] demonstrated consistently increased long-range theta coherence suggesting enhanced connectivity between left posterior areas and the right prefrontal cortex, during episodic memory retention. The increased connectivity demonstrated by [21] was not associated with any change in theta power (amplitude of response), indicating that phase-coupling was a suitable measure of changes in this network. We aimed to extend this finding by examining correlation between time varying levels of spatially averaged PL within each region (frontal and parietal) over time.

Patients with AD are known to have reduced central executive function [97], and this reduction has been associated with decreased fronto-parietal EEG coherence in theta and alpha frequency ranges [16], [20], [21], [58]. Fronto-parietal interaction has already been examined using EEG in true and false memory processing in typical young participants [34]. Fronto-parietal gamma coherence was greater for old (true), than for false or new items. On the basis of the evidence, we postulated that fronto-parietal correlation in theta and alpha PL levels would differ both between false memory compared with true memory, and in adults with MCI compared with age-matched controls.

We chose the right frontal and left parietal regions for investigation for two reasons. Firstly, the literature, as reviewed above, indicates that these are likely to be sites where relevant processing is carried out. The hemispheric encoding/retrieval asymmetry (HERA) model describes the frontal lateralisation of encoding versus retrieval processes, with right frontal involvement in episodic memory retrieval [65], [98]. Indeed, frontal lateralisation for retrieval of episodic memory has been demonstrated using PET studies [63], [65], [72], PET with ERPs [70], repetitive transcranial stimulation (TMS) [99], and neuroimaging studies [76], [90], [100]. The left parietal area was chosen, as verbal information is thought to be maintained in a phonological loop localised within the left parietal area [19], [64], [101], [102], and sub-vocal rehearsal of visually presented words is thought to activate this loop [19], [95].

The second reason for focus on right frontal and left parietal areas, i.e. regions in opposite brain hemispheres, was that one aim in this study was identification of a potential early diagnostic indicator of AD. Imaging and behavioural studies have shown that episodic memories involve interhemispheric interactions, with semantic memory processed mainly on the left [103]. As interhemispheric activity and corpus callosal size have been shown to be reduced in AD [30], [104], this could also contribute to the increase in false memories in AD. By assessing correlation between activity levels in opposite hemispheres, we postulated that the probability of identifying an intergroup difference would increase.

In summary, a review of the literature suggests that neural correlates of true and false memory offer a promising way of differentiating between typical older participants and those with MCI at risk of developing AD. There is considerable evidence that not only performance will differ, but that the differences in neural processing during such a task will be detectable by examining the phase synchrony of right frontal and left parietal activity in the theta and alpha frequency ranges. In this paper, we provide a test of this hypothesis using EMDPL to identify phase synchronies between these brain regions in participants with MCI compared to typical age-matched controls.

Amplitude differences between groups and conditions in the theta and alpha frequencies were also calculated, to explore potentially complementary characteristics of the EEG data that could highlight inter-group differences. While the Hilbert and wavelet transforms have been shown to provide equivalent phase information [105], [106], the differing approaches to time-frequency decomposition offered the possibility of complementary findings by using both approaches. Finally, we performed an ERP analysis to verify that the dataset contained the EEG correlates that would be expected from previous studies [68], [69], [107], [108], [109], [110], [111].

## Methods

### 1. Ethics Statement

Ethical approval was obtained from the University Ethics and Research Committee of the School of Psychology at the University of Reading (Ref. 2006/120/JE for the pilot study and 2006/146/JE for the EEG study) and all participants gave their informed written consent prior to their inclusion in the study.

EEG data were recorded from participants with MCI and age-matched controls using the DRM paradigm, and phase synchrony levels, amplitudes, and ERPs were compared both between groups and between true and false memory processing within groups.

### 2. Experimental Protocol

**Figure 1:**
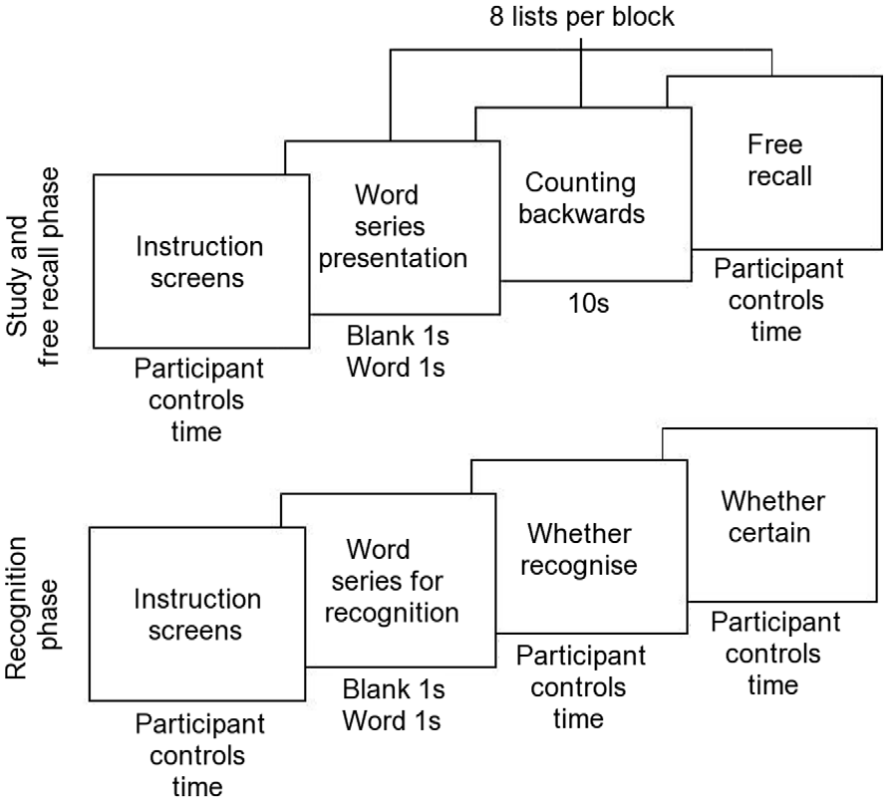
Behavioural paradigm. Experimental protocol followed for the recording of EEG data during performance of the Deese-Roediger-MacDermott paradigm.

The paradigm, summarised in Fig. 1, was based on the DRM word recognition task used by [34]. 24 lists of 10 words were shown serially on a screen, each followed by free recall, and recognition memory was tested using lists of 12 words pertaining to each lure, consisting of 6 words randomly taken from each list, the lures, two of the highest associates of each lure, and three of the lowest associates as foils. In total, 180 words, in their associated groups, were shown for encoding, and 288 were presented in a mixed order for recognition (see Table S1, for word lists). To increase the number of false recalls, no non-associated words were used [34]. To ensure that participants would not see a pattern and realise that the lure was never included in the studied lists, the high and low associates and the lure were presented for 6 of the lists, and the moderate associates were used as the foils in the recognition phase. These lists are referred to as reversed lists. For all lists, high associates were presented first to increase the chance of false memory [34].

Words were taken primarily from the lists provided by [38] and [112]. The selection of lists most likely to produce false memory was informed by [112], in which lists were ranked according to likelihood of producing false memory in the recognition phase. The lists were presented in a mixed order, so that those most likely to lead to false memory were not all together. For those lures used by [38], the associates for the new words in the recognition phase were taken from the lists given. The Florida Word Association Norms [113] were used to select the two highest and three lowest associates from these lists. For those lures not found in the lists by [38], the associates were drawn entirely from the Florida Word Association Norms. Words judged to be more commonly used in American English were excluded, after checking their rankings in the Edinburgh Word Association Norms [114], and the chosen words had an equal forward and backward association with the lure as far as was possible.

Full instructions were given at the beginning and repeated as a reminder at each stage. Participants controlled reading time. As per [34], the trials were completed in three blocks. In each block, participants studied items from 6 regular and 2 reversed lists. Each word was shown for 1000 ms followed by a blank screen for 1000 ms. After list presentation, participants counted backwards aloud for ten seconds as a distraction task and then freely recalled as many words as possible. At the end of each block, the 96 word recognition test was performed, with the 48 studied words and 48 new words, all associated with the lure. Word recognition was chosen to minimise movement artefacts in the EEG recordings, and responses were recorded using a simple button-press. Words were presented for 1000 ms, then participants pressed a ‘yes’ or a ‘no’ button to indicate whether they recognised the word, then pressed a button a second time to indicate whether they were certain or not, in response to ‘Are you certain?’ on the screen. Analysis included only trials with high-confidence responses to ensure that guess rates would not intrude, as per [34]. PL, amplitudes, and ERPs in EEG recorded during trials classified as ‘hits’ (correct recognition of old words) were compared with ‘false alarms (FA)’ (incorrect recognition of new or lure words), as the focus was on true/false effects.

### 3. Participants

A pilot study was performed with two young (mean age 34 years) and two older participants (mean age 71 years), contacted via the Aging Panel recruited from the community by the School of Psychology at the University of Reading. Several adjustments followed the pilot study. In the recognition phase, participants were given control over response time length, since if the next word appeared before a response was made, participants fell behind. The 1 s of EEG recorded during word presentation was used for EEG analysis. The number of response keys was reduced from 4 for recognition and certainty to two, for ‘yes’ and ‘no’ to each question. Instructions were made clearer. Finally, the word list presentation was fixed.

Episodic memory impairment is generally greater in MCI than expected for normal aging [115] and is commonly studied through word recall and recognition tasks [72]. The Consortium to Establish a Registry for AD (CERAD) word learning test provides sub-tests for each measure, and both sub-tests have an established sensitivity to MCI and AD [116], [117]. Delayed recall has been found to be the best discriminator overall when comparing participants with mild AD versus controls [4], [118], [119], [120]. Given the relative success of these tasks in identifying those at risk of AD, immediate and delayed word recall and delayed recognition were measured to contribute to identifying groups of typical older participants and those with MCI for this study.

As for the pilot study, participants for the main study were also identified from the Aging Panel. Typical participants were required to score 28–30 on Mini-Mental State Examination (MMSE) while participants with MCI were chosen from those scoring between 23–28 on this test, in accordance with ICD-10 criteria [121]. Account was also taken of scores primarily in the CERAD delayed recall test and secondarily in the CERAD immediate recall and recognition tests. All participants were right-handed. Potential participants were contacted by telephone initially and with their agreement, were sent an information sheet and consent form. 22 participants were recruited, with 11 per group. One participant in the MCI group felt unwell on the testing day and did not complete any blocks, so was excluded from the analysis, and a second participant developed discomfort in one hand in the final block, so completed only two of the three testing blocks. These data were included.

### 4. EEG Recording

Participants sat in a comfortable chair, 57 cm from the screen and with the keyboard within easy reach. EEG was recorded from a 129 sensor net (128 channels), with an Electrical Geodesics, Inc. amplifier and Net Station software. The experimental paradigm was presented using ePrime stimulus presentation software.

Following recording, the data from the recognition phase were bandpass filtered between 0.5 and 100 Hz using a finite response filter with a 2 Hz roll-off. The data were segmented into epochs in ePrime during word presentation for recognition, then exported into Matlab.

### 5. Electrode Selection

In EEG/MEG analysis, particular brain areas are frequently chosen on the basis of well-recognised findings in the literature in order to reduce the vast number of possible comparisons to be made [16], [19], [21], [58], [122], [123], [124]. Given the extensive evidence for right frontal and left parietal involvement in memory retrieval, these regions were selected for region-of-interest analysis. Due to variation between studies in the sensor nets used for EEG recording, it was not possible to select electrodes identical to those used in the relevant studies but rather was necessary to select corresponding electrode locations. We chose electrodes whose locations corresponded most closely with the locations identified in other studies to be involved in similar memory processing tasks. With the sensor net we used, 4 electrodes from each area adequately covered the relevant regions.

**Figure 2:**
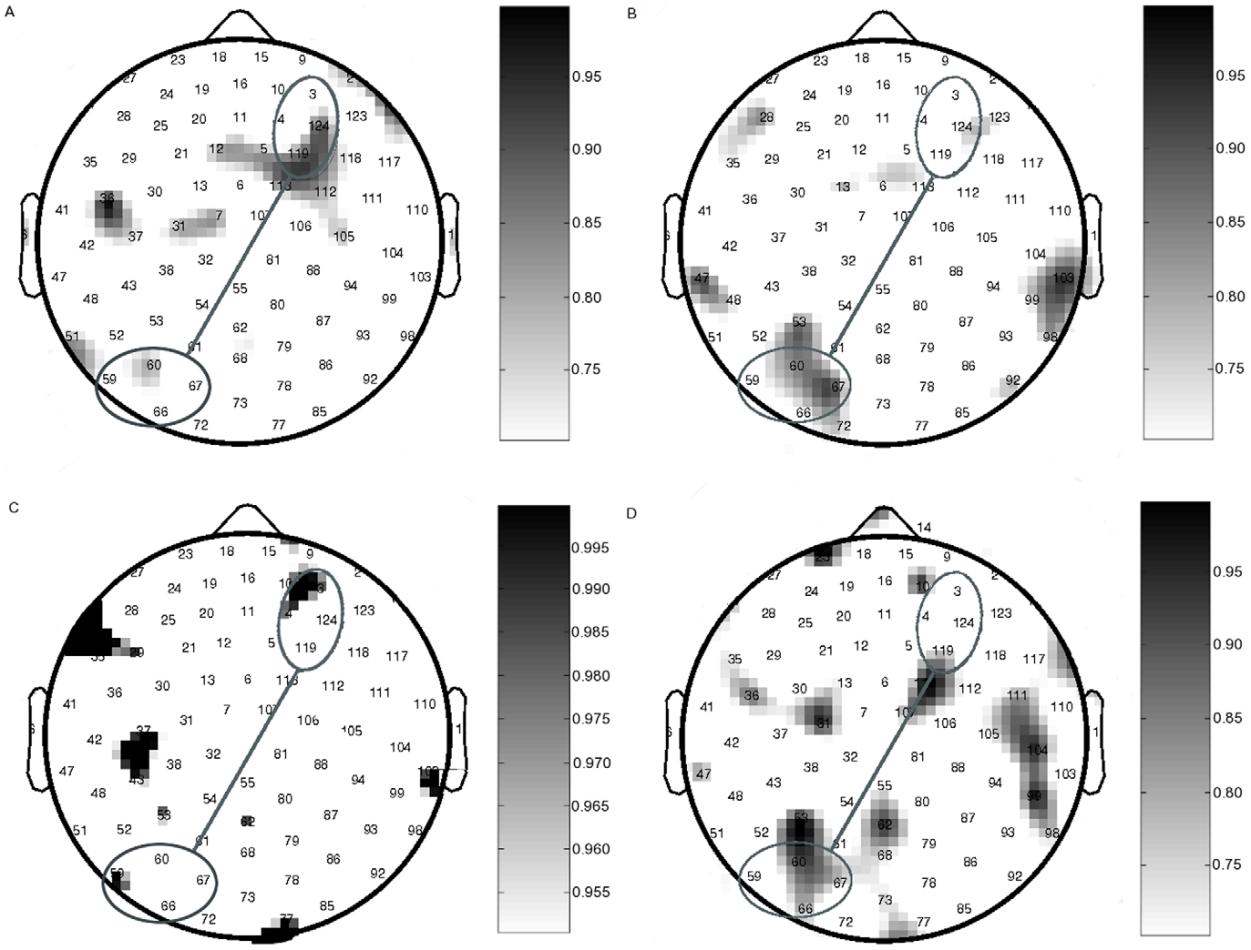
Spatial localisation of ERP and phase alignment differences between hits and FA in typical participants. The right frontal and left parietal electrodes chosen for study are highlighted in the Electrical Geodesics Inc. Sensor Net used for recording EEG. A. ERP differences in the first 250 ms post-stimulus. B. ERP differences 400–650 ms post-stimulus. C. Mean theta phase alignment differences in the first 250 ms post-stimulus. D. Mean alpha phase alignment differences 150–400 ms post-stimulus. The colourbar represents p-values determined using a two-sample T-test.

In order to confirm that the areas chosen were indeed relevant in our data set, we calculated post-stimulus ERP differences between hits and FA in the typical group in all 128 electrodes. We also calculated post-stimulus theta and alpha phase alignment in all 128 electrodes, in order to include a phase-based approach. (As discussed in the introduction, we chose to explore phase synchrony in detail in this work, given the evidence for its role in cognition in general and in memory processing in particular.) Details of the methods applied are in Appendix S1. All 8 electrodes chosen from the literature showed a difference in activity during hits versus FA, supporting their use in the study (Fig. 2).

### 6. Data Pre-Processing

#### 6.1 Artefact removal

As adding the additional instruction of avoiding blinking during trials could impair task performance in participants with MCI, all participants were simply asked to avoid excessive movement. Common in EEG studies is to inspect data visually and reject trials with clear artefacts [14], [125], [126]. This was not possible here, as the vast majority of the trials contained ocular artefacts.

Blinks and eye movements have a different temporal structure when compared with electrocortical activity, therefore temporal decorrelation separation (TDSEP) provided a straightforward method of removing this activity [127]. TDSEP is a type of independent component analysis (ICA), in which signals comprising contributions from a mixture of sources are separated by maximising the temporal independence of the components [128], [129].

**Figure 3:**
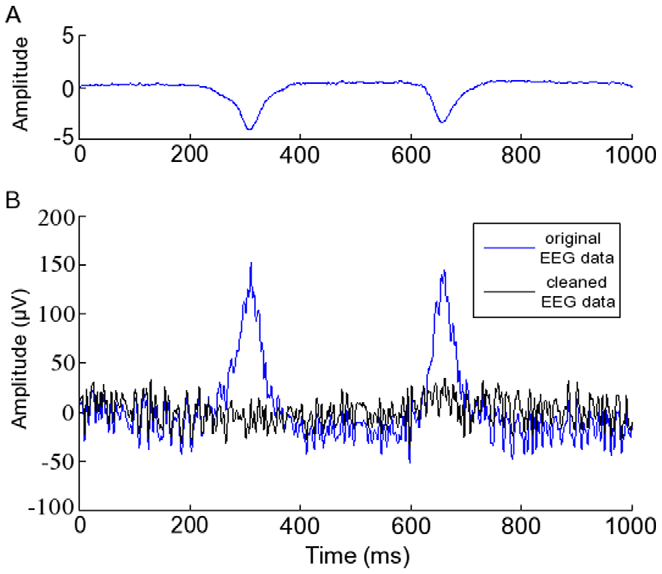
Artefact removal. Illustration of the removal of two blink artefacts using temporal decorrelation separation independent component analysis. A: the component which contained the blink artefacts for one trial. B: the data before and after removal of the latter component.

TDSEP was applied to each trial, and the independent components (ICs) containing the ocular data were removed by visual inspection. The corresponding column of the mixing matrix was set to zero when the ICs were back-projected to the scalp electrodes for reconstruction of the data without artefacts. Fig. 3 provides an illustration of a blink artefact (Fig. 3A) and its subsequent removal from the signal recorded at one electrode (Fig. 3B). Following removal of ocular artefacts, visual inspection was used to exclude any trials clearly contaminated by artefacts from other non-cortical sources.

#### 6.2 Volume conduction

The signals recorded at the scalp surface comprise contributions from multiple cortical sources, which are linearly combined during conduction through the brain parenchyma, cerebrospinal fluid, skull, and scalp. As a result, spurious synchrony can be detected between adjacent electrodes. Laplacian transformation of the raw EEG data not only addresses the problem of choosing a montage [10], [130], [131], which can affect phase synchrony measurement [132], [133], but it also goes some way to removing the effects of volume conduction [10], [131].

A frontal group and a parietal group of electrodes were selected for analysis. In order to reduce volume conduction further, while preserving phase synchrony, a second TDSEP was applied to each group [10], [134]. In summary, the raw data, cleaned of blink artefacts by identifying components of the signals containing blink data using TDSEP, were spline Laplacian transformed, and then TDSEP was applied to the clean, Laplacian-transformed signals. The subsequent analyses, including EMDPL, bandpass filter and wavelet based phase synchrony, phase alignment, amplitude analyses, and ERP calculation were then applied to the resulting components.

### 7. EMDPL

The first stage of EMDPL is performance of EMD. The Matlab code used to implement EMD was adapted from that used by [135], [136]. The initial step is to identify the extrema of each of the pair of signals between which synchrony is to be sought, then to find the upper and lower envelopes by connecting the maxima and minima respectively using cubic spline interpolation. The mean of the envelope of the signal is then subtracted, and the component found is assessed to determine whether it fulfils the two criteria by which an intrinsic mode function (IMF) is defined. The first is that the mean of the envelope must be zero. The threshold used here for acceptable proximity to zero was 0.001. The second criterion is that the number of extrema must differ at most by one. Once an IMF is found, it is subtracted from the signal, and the process is repeated until a stopping condition is reached. This condition requires that each IMF must differ from the previous one according to a mean squared error stopping criterion, which is empirically set at 10e^−5^. Furthermore, each IMF must contribute at least 5% of the variance of the signal [9].

The definition of IMFs allows calculation of instantaneous features of a signal *X_t_* using the Hilbert transform (HT) (Eq. 1),
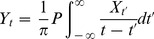
(1)where *Y_t_* is the HT of *X_t_*, *P* indicates the Cauchy principal value of the integral, and *t* is time [46]. Using *Y_t_* as the imaginary component, an analytic signal *Z_t_* is formed, (Eq. 2),
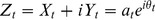
(2)where at is the series of instantaneous amplitudes when the analytic signal is represented in polar coordinates, and θt are the instantaneous phases. Instantaneous frequency ft may be derived, as the rate of change of the instantaneous phase (Eq. 3).



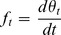
(3)PL is sought pairwise between all possible combinations of the IMFs from the signal pair in question. It is defined in a statistical sense as a constant value of the difference between the series of instantaneous phases over time, as follows [137],
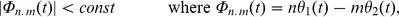
(4)where Φ*_mn_* is the relative phase, *θ_1_* and *θ_2_* are the phase series from each signal, *t* is time, and *n* and *m* are integers. The single-trial phase locking value (SPLV) is calculated using Eq. 5.
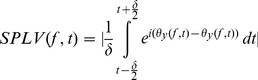
(5)where the phases of signals x and y are θx and θy, at each frequency f and time t, and δ is the time window over which the SPLVs are calculated [138]. In this study, 350 ms temporal windows were used for SPLV calculation [9]. In order to ensure that the results would not be influenced by PL outside the 1 s during which participants looked at the presented word, the EMDPL spectra were plotted from 200 to 800 ms. Note that as SPLVs are calculated over time for each trial, the number of trials for a given condition does not affect the phase locking values.

Statistically significant episodes of PL were identified using phase-scattered surrogate data derived from the IMFs in question, and significant phase synchrony was localised in time and frequency on the EMDPL spectrum [9]. A 95% significance cut-off was applied to phase scattered surrogate data for each IMF pair in order to determine when PL occurred more strongly than that which would be found by chance between similarly constructed signals with independent phases [9]. Any PL with a value greater than 95% of background PL was plotted in bins on the EMDPL spectrum for the given electrode pair. It should be highlighted that the use of phase-scattered surrogate data is actually a step in the EMDPL method itself. IMFs are frequency-modulated, with frequency and also bandwidth varying over time, and each trial is decomposed uniquely, adapting to individual data content. We have shown previously that background phase locking levels are dependent on bandwidth, which means that when a comparison is made between synchrony levels in two different signals, account must be taken of the bandwidth of a given component at the time of interest [9]. As a result, it does not make sense, when EMD is used, to calculate a PLV over trials directly as per [12]. We addressed this problem by generating phase scattered surrogate data for each IMF individually, calculating single-trial PLVs across time [138], and defining synchrony in a binary fashion, as an SPLV for a given time window being at a level greater than that in 95% of pairs of phase-scattered surrogates from the given IMF. A synchrony index is then determined at a particular time and frequency for a given condition by finding a mean across trials, a step analogous to the calculation of PLV across trials. In the next section, we describe the statistical analysis involved in comparing groups and conditions.

Following calculation of the EMDPL spectra, the grand EMDPL spectrum was then created by summing the EMDPL spectra for the 6 possible combinations of 4 electrodes in the right frontal network, across all trials for each participant, and similarly for the left parietal network. These were then in turn summed over participants to yield two EMDPL spectra, which included all trials for all participants, one for the frontal networks, and one for the parietal networks, for hits and FA in typical participants, and the same for those with MCI. In total, 8 such EMDPL spectra were obtained, one for each combination of a region, participating group, and experimental response.

### 8. Statistical Analysis

#### 8.1 Two sample two-dimensional Kolmogorov-Smirnov test

The EMDPL spectra for normal participants and those with MCI were compared during hits and FA using two sample two-dimensional Kolmogorov-Smirnov (2D K–S) tests, to determine significant differences between the distributions of PL values in the different participant groups and response types. This statistical test was chosen as account is taken both of frequency and time simultaneously. This extension to the classical one-dimensional Kolmogorov-Smirnov test was implemented here in Matlab [139], [140]. Details of the algorithm were taken from [141].

In order to establish whether two data sets were drawn from the same 2D distribution, both sets are first plotted together. The 2D K–S test then involves using each data point from one sample as the origin of the plot, which is then divided into four natural quadrants. In each quadrant, the difference between the number of points from sample one and the number from sample two is calculated and normalised. This procedure is then repeated using each data point from sample two as the origin. The mean is taken of the largest difference found using data points from sample one as the origins and those using points from sample two. This quantity is termed the D-statistic. The significance levels for the D-statistic can be approximated as follows [141]:
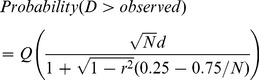
(6)where:



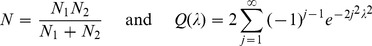
(7)
*N* is the number of samples. *r* ^2^ is a sum of *r*_1_^2^ and *r*_2_^2^, where *r*_1_ is the Pearson’s correlation coefficient between the *x*- and *y*-coordinates of sample one, and *r*_2_ is the equivalent for sample two. Eq. 6 gives the probability that the null hypothesis is true, and both samples are drawn from the same distribution.

Monto Carlo simulations have been used to demonstrate that this approach provides a distribution-free test [139]. Furthermore, it takes account of the sample sizes and also the degree of correlation of the data points [139].

#### 8.2 Two sample T-test

The particular times and frequencies at which the time-frequency PL pattern differed significantly between study conditions or participant groups were then evaluated using the two sample T-test, implemented in Matlab. PL levels were averaged over 2 Hz frequency windows with 1 Hz overlap and 9 ms time windows for each participant, for group and condition contrasts, and Bonferroni correction was applied for multiple group/condition/frequency comparisons. The figures show differences significant at p<0.05 after Bonferroni correction.

#### 8.3 Pearson’s correlation coefficient

The degree of correlation between frontal and parietal time varying PL levels was calculated in the same frequency windows over trials and subjects, applying 100 ms time windows with 25 ms overlap, using Pearson’s correlation coefficient [141]:
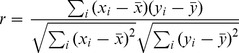
(8)
*r* varies between −1 with complete negative correlation and 1, with complete positive correlation. 0 implies no correlation.

The significance of *r* was determined using the *t*-distribution, calculated using Eq. 9 [142],
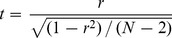
(9)where *N* is the number of pairs of samples, and *N*-2 gives the number of degrees of freedom. The figures show results significant at p<0.05 and below after Bonferroni correction for groups/conditions/frequencies compared.

### 9. Traditional Phase Synchrony Analyses

#### 9.1 Bandpass filter based phase synchrony

Phase synchrony was also calculated following application of bandpass filters, both in order to validate the EMDPL analysis and to demonstrate the advantages provided by EMDPL. First, a 4–8 Hz 4^th^ order Butterworth filter was applied, using commonly defined limits for the theta frequency range, to investigate whether the phase synchrony detected using EMDPL could indeed be identified using a standard method. Secondly, a 4^th^ order Butterworth filter was applied using a range of frequency cut-offs identified in the literature, as listed in the introduction, as denoting the theta frequency range. The latter was focused on an episode of theta phase synchrony identified using EMDPL. The same statistical analyses applied following EMDPL were also used here.

#### 9.2 Wavelet based phase synchrony

We also carried out phase synchronisation analysis following a wavelet time-frequency decomposition, as is commonly applied in neuroscience. The signal was convoluted with the wavelet basis function, which was dilated to evaluate frequency content and translated over time [143]. Wavelet calculation was carried out using a modified version of the Matlab toolbox by [144]. The complex Morlet wavelet, a complex sine wave modulated by a Gaussian, was used as the mother wavelet:
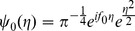
(10)where *f_0_* was the nondimensional frequency (the number of cycles in the wavelet), and *η* was the nondimensional time parameter of the shift divided by the dilatation. Six cycles were used per wavelet [9], [145], [146]. Following the wavelet transform, amplitudes and phases were extracted. Further details of this well-known approach may be found in [9], [12], [143], [146]. The significance assessment was analogous to that following EMDPL, including application of a Bonferroni correction.

### 10. Amplitudes

Significant differences in amplitude following time-frequency decomposition were also sought. Time-frequency decomposition was carried out using EMD, and an alternative time-frequency decomposition was also performed using the wavelet transform in order to provide a comprehensive characterisation of the groups/conditions.

Significance was calculated for amplitudes using the same sliding time window as for EMDPL, but no Bonferroni correction was used.

### 11. ERPs

The data were first filtered using a 1–30 Hz 4^th^ order Butterworth bandpass filter then averaged over trials for each participant. A mean ERP for each group/condition was then calculated across participants for each condition, and a 100 ms baseline was subtracted. Statistical analysis was carried out using T-tests to compare mean ERP amplitudes between groups and conditions over sliding windows of 50 ms. The data were filtered between 1–12 Hz for the purpose of displaying the ERP waveforms, but the 1–30 Hz filtered data were used for the statistical analysis. (A 12 Hz filter is often applied to provide clear visualisation (e.g. [147], [148]). However, it is not deemed acceptable to use such a filter for ERP quantification [149]. For statistical analysis, ERPs are commonly filtered using a 30 Hz filter (e.g. [147], [150], [151]). 20 ms time windows were used to calculate amplitudes to assess latency of the P300 component, which is thought to be related to reactivation of memory traces. The time of the P300 peak was taken as the central time in the highest amplitude 20 ms time window between 200 and 400 ms in comparing latencies for hits versus FA in the normal group. Similarly, to compare latencies for hits in the typical group versus the group with MCI, the highest amplitude in a time window between 300 and 500 ms was determined, to account for longer latencies in the MCI group. The ERPs were calculated for all 8 electrodes, but as the ERPs were only being considered as an illustration that the data contain expected relationships well-recognised in the literature, we focused on the parietal ERP in which inter-condition and inter-group differences were significant.

## Results

### 1. Behavioural Findings

The participant selection criteria and behavioural test scores before inclusion in the study are shown in Table 1. The typical and MCI groups were matched for age, years of formal education, and gender (T-test p-value >0.05). The groups differed significantly, however, in performance in the MMSE (p<0.00005) and the CERAD delayed recall test (p<0.00005), which were the primary measures used to determine typical and MCI group allocation. In the immediate recall and recognition tests, participants in the typical group achieved higher scores, but the difference did not reach statistical significance.

**Table 1:** Parameters for typical and MCI participant groups.

Test	Typical	MCI	p-value from
	Mean (SD)	Mean (SD)	T-test
Age	68.2 (7.1)	71.6 (3.9)	0.1915
Years formal education	13.5 (2.6)	14.9 (5.5)	0.4711
Gender (F:M)	1.5 (0.5)	1.6 (0.5)	0.8127
MMSE	28.8 (0.8)	26.9 (0.9)	<0.00005
Immediate recall^a^	0.720 (0.097)	0.713 (0.089)	0.8655
Delayed recall^a^	0.845 (0.113)	0.450 (0.190)	<0.00005
Recognition^a^	0.995 (0.015)	0.950 (0.091)	0.1193

a Note: CERAD recall and recognition scores are normalised.

Tables 2 and 3 show the behavioural results for the DRM paradigm, carried out during EEG recording. The typical group had a significantly higher correct free recall rate (p<0.01) than the MCI group, while adults with MCI produced the critical lure significantly more frequently (p<0.05) than the typical group. In the recognition phase, while the participants from the typical group achieved more hits and fewer FA than the MCI group, the difference between groups was not statistically significant, indicating that the impairment in the MCI group was indeed mild. It can be seen that there was no statistically significant difference between the groups’ performance in either the CERAD or the DRM word recognition tests. The significant between-group difference in the DRM free recall, when none was found on the CERAD immediate free recall, might be accounted for by increased delay due to the distractor task in the DRM test.

**Table 2:** Free recall using the DRM paradigm.

Free Recall (%)	Typical^a^	MCI^b^	p-value from
	Mean (SD)	Mean (SD)	T-test
Correct recall	54.30 (13.52)	39.00 (7.64)	<0.01
Critical lure	19.53 (9.50)	32.14 (15.65)	<0.05

a Total of 1980 words presented to typical participants for encoding.

b Total of 1740 words presented to participants with MCI for encoding.

**Table 3:** Word recognition using the DRM paradigm.

Recognition (%)	Typical^a^	MCI^b^	p-value from
	Mean (SD)	Mean (SD)	T-test
Hits	49.9 (4.9)	48.9 (9.0)	0.5216
False alarms	15.9 (10.0)	18.6 (9.5)	0.6712

a Total of 3168 words presented to typical participants for recognition.

b Total of 2784 words presented to participants with MCI for recognition.

#### 1.1 Reaction times

Reaction times (RTs) from the DRM recognition test were compared between conditions and groups using a two sample T-test. In both typical participants and those with MCI, the mean RTs were significantly longer for hits than for FA (typical: 2631 ms vs. 2036 ms, p<0.04, MCI: 2372 ms vs. 1989 ms, p<0.004). RTs both for hits and for FA were significantly longer for the typical group than for the MCI group (hits: 2036 ms vs. 1989 ms, p<0.00021, FA: 2631 ms vs. 2372 ms, p<0.0422).

The analysis was performed again after removing all RTs >5000 ms, as some RTs were over 10 s, and it was deemed possible that the participants had paused for some extraneous reason during those trials. In typical participants, 2.9% of hit responses and 5.1% of FA responses occurred over 5 s after the stimulus, as opposed to 2% of hits and 0.83% of FA in the MCI group. Again, RTs for hits were significantly longer than for FA both in typical participants (p<0.04) and in MCI (p<0.04). RTs remained slower for the typical group compared with those with MCI for hits (p<0.0014) and for FA (p<0.04). The analysis was also performed using median RTs, both including and excluding RTs over 5 s. The same pattern with respect to conditions and groups remained statistically significant.

#### 1.2 Certainty

Significantly more of the hits were rated as certain in the typical group, compared with the MCI group (91.7% vs. 83.1%, p<0.00001).

### 2. EMDPL

The time-frequency distributions of SPLVs which reached significance (5% threshold compared with the surrogate data) are termed the EMDPL spectra. The mean EMDPL spectra over trials and participants for the right frontal and left parietal regions were first compared for both groups for hits and FA using the 2D K–S test. The test was applied to the entire post-stimulus time period of interest (200–800 ms) and over the whole frequency range of interest, which included theta and alpha activity from 4–12 Hz. The p-values were corrected for multiple comparisons (groups/conditions) and are shown in Tables 4 and 5.

**Table 4:** Two sample two-dimensional Kolmogorov-Smirnov test comparing time-frequency distributions of phase locking values in frontal and parietal networks.

Hits vs. False Alarms	Typical^a^	MCI^a^
Frontal	<0.01	<0.01
Parietal	<0.05	<0.05

a p-values given after correction for multiple comparisons.

**Table 5:** Two sample two-dimensional Kolmogorov-Smirnov test comparing time-frequency distributions of phase locking values in frontal and parietal networks.

Typical vs. MCI	Hits^a^	False alarms^a^
Frontal	<0.005	0.1059
Parietal	<0.05	<0.05

a p-values given after correction for multiple comparisons.

Significant differences with a p-value <0.01 were identified in 3 of the comparisons of SPLVs within frontal regions: hits versus FA in typical participants (p-value <0.01), hits versus FA in participants with MCI (p-value <0.01), and for hits in typical versus participants with MCI (p-value <0.005).

**Figure 4:**
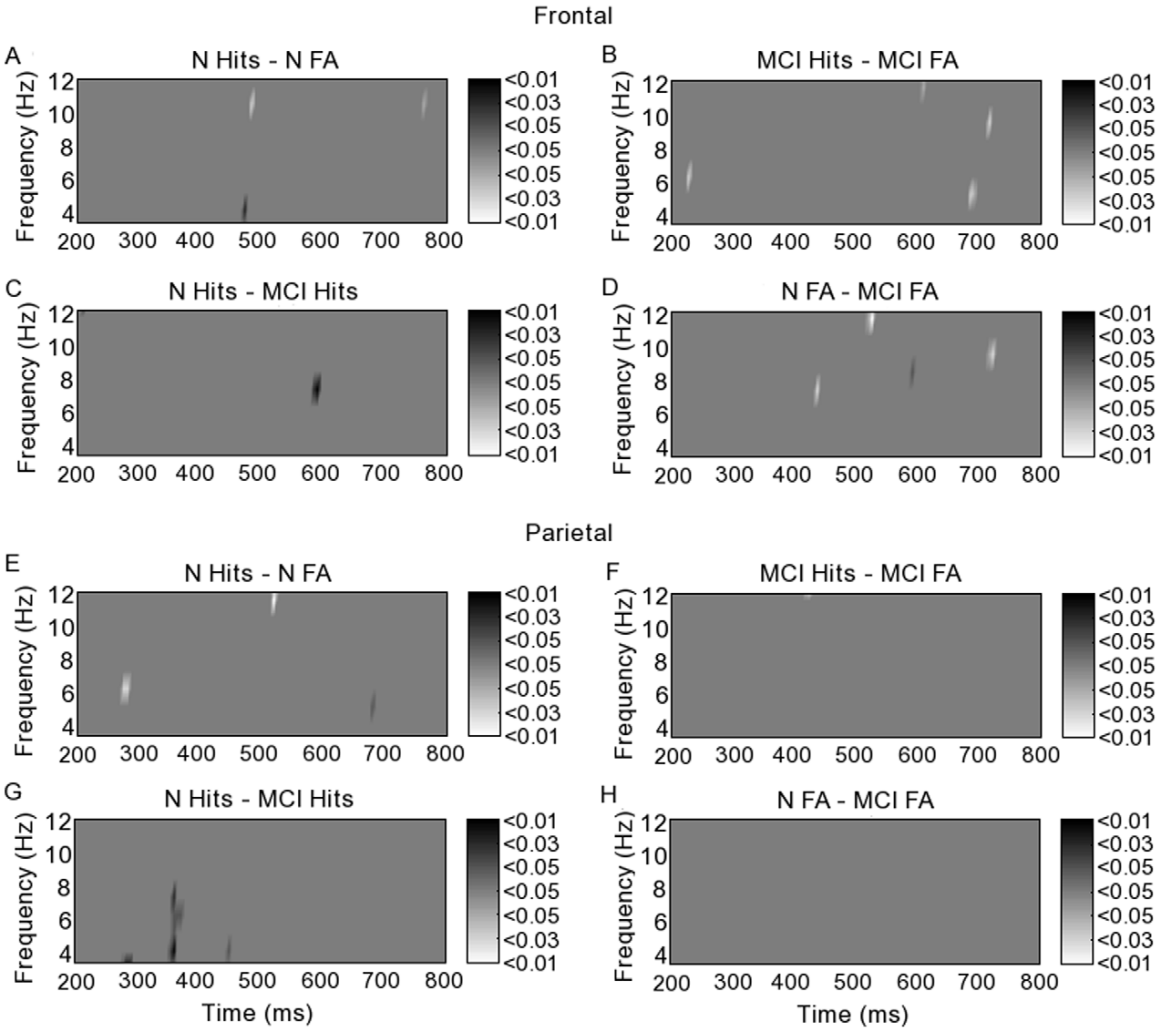
Phase locking differences between groups and condition, calculated using empirical mode decomposition phase locking analysis. p-values corrected for multiple comparisons are shown using the greyscale. Zero represents no significant difference, the dark scale is for when the first named group and condition is significantly greater than the second, and the light scale is for when the second group/condition is significantly greater.

The EMDPL spectra were then compared using a two sample T-test, with sliding time and frequency windows, in order to identify the time and frequency of occurrence of any significant difference in PL levels between groups or conditions. Significant differences are shown in Fig. 4, in which p-values are plotted. Black indicates the PL in the first condition was significantly greater than in the second, and white indicates that the PL in the second condition was significantly greater than in the first.

In typical participants, there was significantly greater (corrected p<0.01) lower theta (4–5 Hz) frontal PL at 475 ms in hits than in FA (Fig. 4A). The finding was absent in the MCI group (Fig. 4B). In participants with MCI, significantly more theta PL was found in FA vs. hits both earlier, at 230 ms (corrected p<0.03), and later, at 700 ms (corrected p<0.01).

In upper alpha (10–12 Hz), there was significantly greater frontal PL in FA than hits in the typical group (corrected p<0.01) at 475 ms, the time at which theta PL was significantly greater in hits (Fig. 4A). There was significantly greater frontal upper theta PL (6–8 Hz) at 600 ms in the typical group than in the MCI group, more markedly for hits (corrected p<0.01) (Fig. 4C) but also for FA (corrected p<0.05) (Fig. 4D). At 750 ms, there was significantly more alpha PL for FA than for hits in both the typical group (corrected p<0.03) and more so in the MCI group (corrected p<0.01). In the typical group, this PL took place around 40 ms earlier than in the MCI group.

Significantly greater theta (4–8 Hz) PL (corrected p<0.01) was found in the parietal network at 350 ms in the typical group during hits compared with the MCI group (Fig. 4G). In the typical group, significantly greater theta (6–8 Hz) PL (corrected p<0.01) took place at 280 ms for FA than for hits (Fig. 4E). There were no significant differences in PL in the theta or alpha frequency ranges in the MCI group for hits versus FA nor for FA in the typical versus MCI groups (Fig. 4F and H).

### 3. Fronto-parietal Correlation

**Figure 5:**
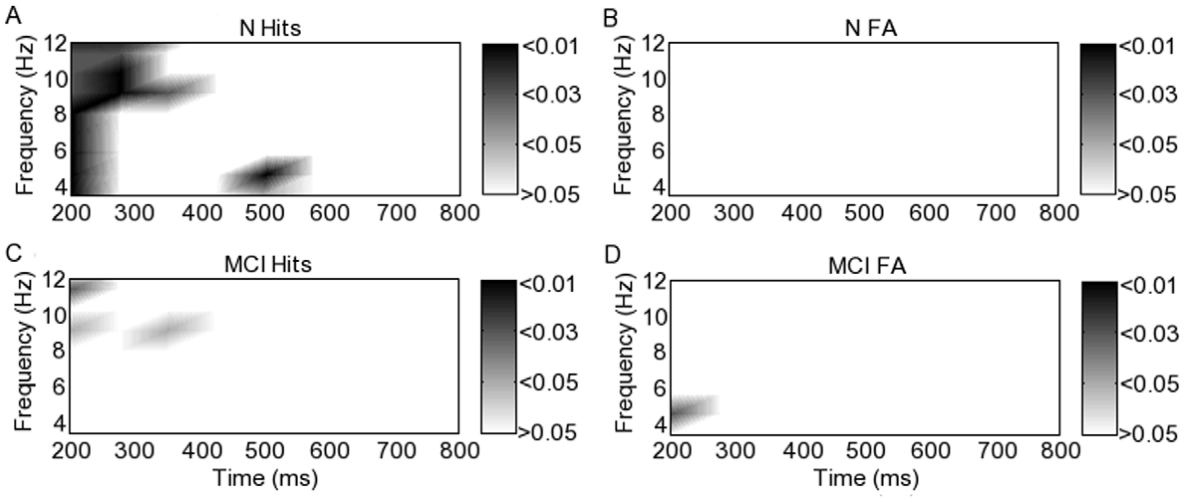
Fronto-parietal correlation between levels of phase locking calculated using empirical mode decomposition phase locking analysis. p-values corrected for multiple comparisons are shown in greyscale for typical and mildly cognitively impaired groups during hits and false alarms.

Correlation between spatially averaged frontal and parietal PL fluctuations was significant at 450–550 ms (corrected p<0.01) during hits in the typical group in lower theta (4–5 Hz) (Fig. 5A). This correlation was neither seen during hits in the MCI group (Fig. 5C) nor during FA in either group (Fig. 5B and D). During hits in both groups, significant fronto-parietal PL correlation was seen at 200 ms (corrected p<0.05) and again at 300 ms in lower alpha (8–10 Hz) (Fig. 5A and C), though more markedly in the typical group (corrected p<0.01), and also at 200 ms in upper alpha (10–12 Hz) in both groups, persisting for longer in the typical group (p<0.01) than in the MCI group (p<0.03).

### 4. Traditional Phase Synchrony Analyses

#### 4.1 Bandpass filtered based phase synchrony analysis

**Figure 6:**
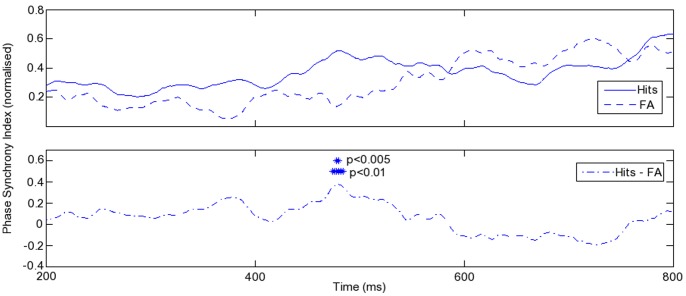
Phase synchrony calculated following 4–8 Hz bandpass filtering during hits and FA in typical participants. A. Single trial phase locking values (SPLVs) averaged over trials and subjects in hits and in false alarms (FA). B. The difference between SPLVs averaged across trials in hits versus FA. Asterisks indicate the time period in which the difference was found to be statistically significant.

**Figure 7:**
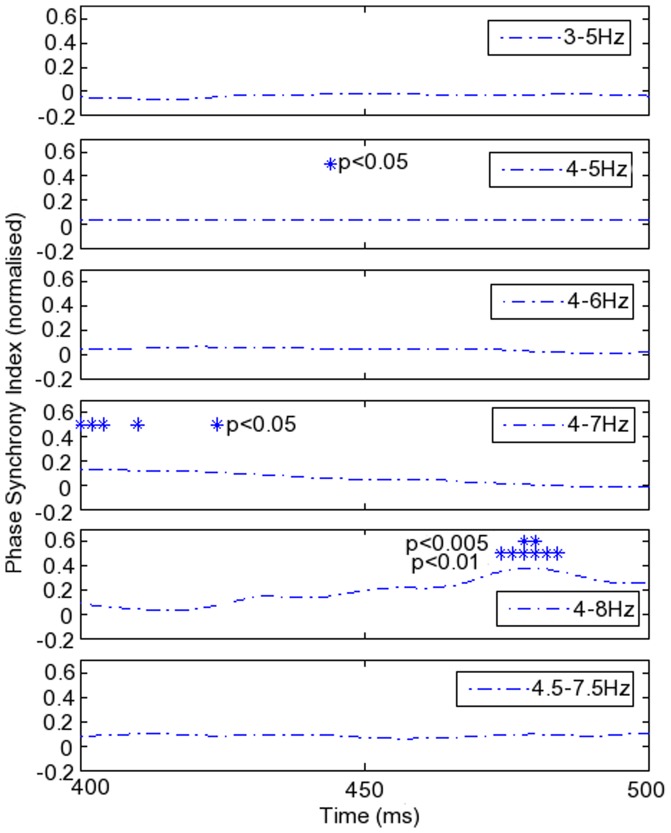
Varying bandpass filter corner frequencies in the theta frequency range. Differing frequency cut-offs were identified in the literature. Both the detection and the time localisation of significant differences in phase synchrony between conditions were affected by the a priori choice of bandpass filter corner frequencies. Note that the scale for the phase synchrony index is the same for each panel to facilitate direct comparison. As a result, the peak differences found in the second and fourth panels from the top are only to be seen by reference to the asterisks indicating the timing of a significant difference.

Phase synchrony was detected at the same times and frequencies as identified using EMDPL, but the findings were dependent on the precise filter cut-offs applied, as illustrated in Figs. 6 and 7, in which hits and FA are compared in the typical group. The greater theta synchrony in hits than in FA identified just before 500 ms using EMDPL was detected and found to be significant when a 4–8 Hz bandpass filter was applied (Fig. 6), supporting the EMDPL findings. However, application of different theta filter cut-offs taken from the literature demonstrated that the choice determines not only the timing of synchrony detected but also whether it is detected, confirming findings in [10], where EMDPL was contrasted with standard approaches to phase synchrony calculation using both data synthetically constructed for the purpose and EEG data recorded during a simple motor task (Fig. 7).

#### 4.2 Wavelet based phase synchrony analysis

**Figure 8:**
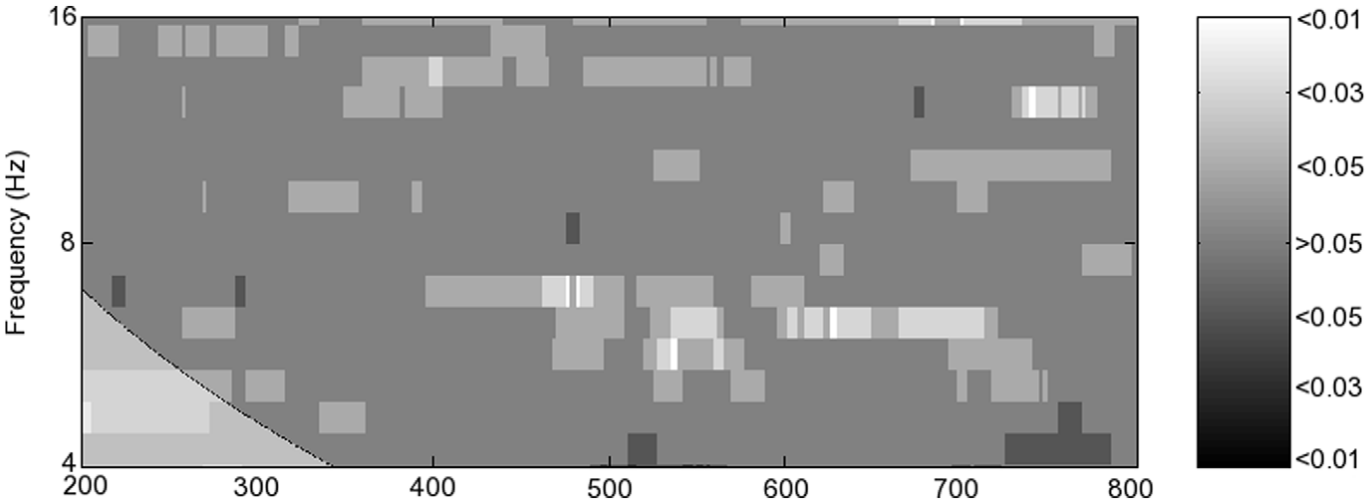
Phase locking differences between hits and FA in participants with MCI calculated following wavelet decomposition. p-values corrected for multiple comparisons are shown using the grayscale. Zero represents no significant difference, the light scale is for hits > FA, and the dark scale is for FA > hits. The light grey area in the lower left corner of the upper panel represents the region lying in the so-called cone of influence, where edge effects occur, making results unreliable.

Again, following wavelet based phase synchrony analysis, the inter-group and condition phase synchrony differences found using EMDPL were detected, suggesting that the findings reflect the content of the data rather than being an artefact of EMDPL. However, when wavelets are used for time-frequency decomposition, there is a trade-off between localisation in time and in frequency, such that time localisation is sacrificed at lower frequencies. The findings are illustrated in Fig. 8. The greater synchrony in hits than FA in the MCI group was again detected in theta between 200 and 300 ms and between 600 and 700 ms, as well as in alpha between 700 and 800 ms (Fig. 8). p-values are shown following correction for the same multiple comparisons as accounted for using EMDPL. The presence of these episodes was thus confirmed following a time-frequency decomposition unrelated to EMD, strengthening the findings. However, as expected, in these low frequency ranges, the time-frequency trade-off is evident, with reduced localisation in time.

### 5. Amplitudes

**Figure 9:**
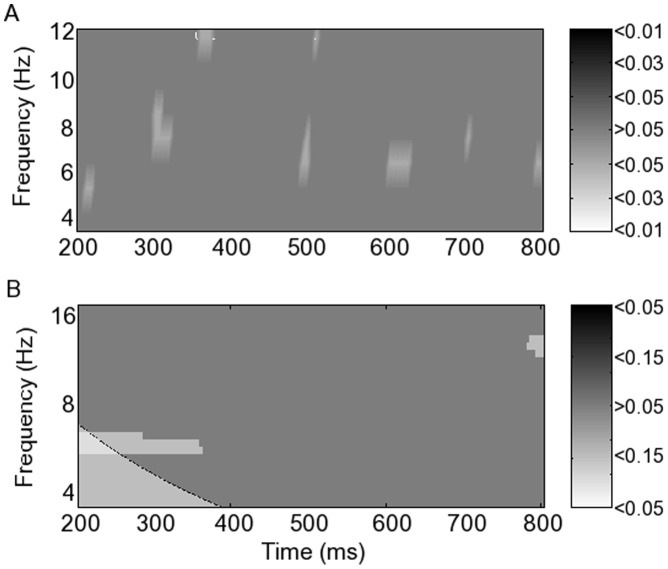
Difference between amplitudes calculated following empirical mode decomposition and wavelet decomposition. p-values are shown for the 4^th^ parietal electrode in hits vs. false alarms in the typical participant group. Zero represents no significant difference, the dark scale is for hits > false alarms, and the light scale is for false alarms > hits. The light grey area in the lower left corner of the upper panel represents the region lying in the so-called cone of influence, where edge effects occur, making results unreliable.

Amplitude differences were calculated following EMD. Note that although the scale in the figure matches that used in the figures showing PL, the latter are corrected p-values. A significant amplitude difference was found only in the 4^th^ parietal electrode between hits and FA in typical participants in theta (Fig. 9A). The difference, however, was only significant at p<0.05 in the absence of Bonferroni correction. Given the absence of significant inter-group/condition differences, amplitude differences were also sought following wavelet decomposition (Fig. 9B) Theta amplitude at 300 ms was also found to be greater in FA than in hits following wavelet decomposition, but only at p<0.15. Indeed no amplitude differences were significant following wavelet decomposition, even without correction. Moreover, the time-frequency trade-off when wavelets were used meant that time localisation in this low frequency range was reduced, with the episode beginning before 200 ms and continuing to 360 ms. When EMD was applied, the episode was localised to around 290–310 ms. The beginning of the episode, as detected by wavelets, was affected by the cone of influence, the border region in which wavelet calculation is unreliable, but appears to start at least as early as 220 ms. Note that the frequency scales correspond with the frequencies at which amplitudes are most appropriately calculated for each method, in particular to reduce redundancy with the wavelets.

### 6. ERPs

**Figure 10:**
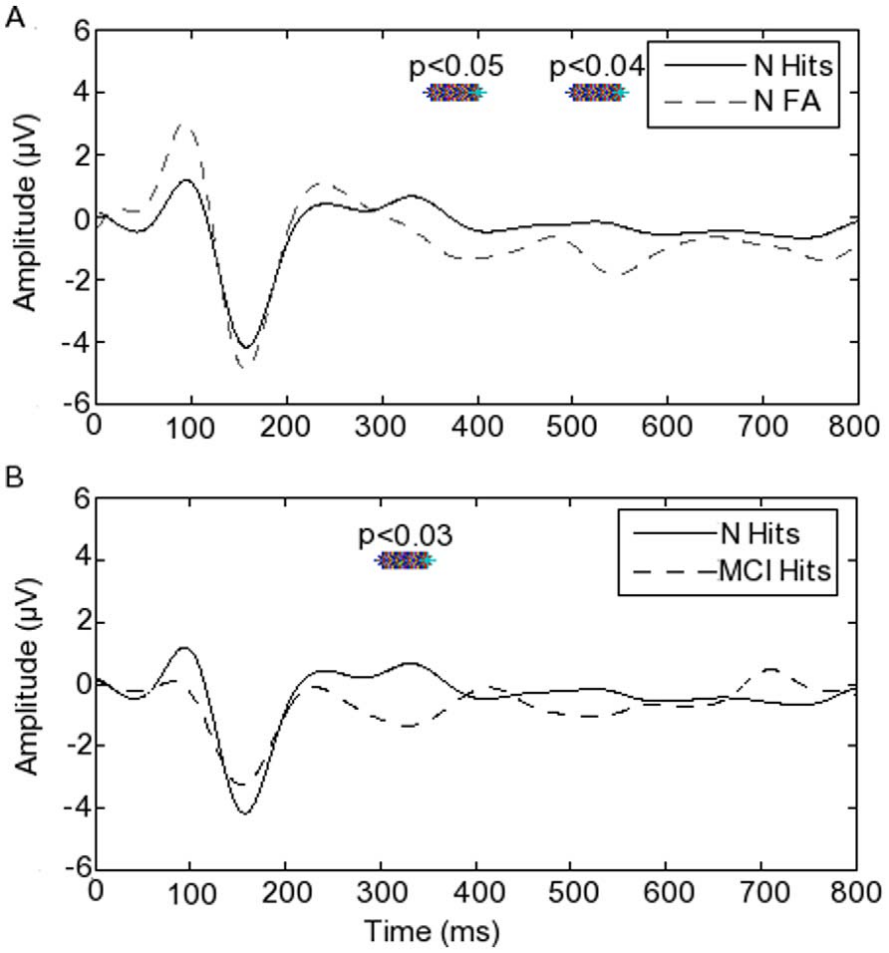
Left parietal event-related potentials differing between conditions and groups. Asterisks mark the time during which there was a statistically significant difference between the event-related potentials shown, with the relevant p-values (uncorrected).

ERPs during hits and during FA in the typical group are shown in Fig. 10A, and during hits in the typical group and in the MCI group in Fig. 10B. The amplitude of the ERP during hits was significantly greater at 340–400 ms (p<0.05) and again from 480–550 ms (p<0.04) than during FA in the typical group (Fig. 10A). The ERP from 290–360 ms during hits in the typical group was significantly more positive than for the group with MCI (p<0.05) (Fig. 10B). The latency of the P300 peak in the typical group during FA was shorter than that for the MCI group, though this difference did not reach statistical significance (p<0.09). The P300 latency for hits in the MCI group was significantly longer (p<0.009) than for hits in the typical group.

## Discussion

The DRM paradigm was used to explore the neural correlates of veridical and false memory in participants with MCI and age-matched controls, and analysis of functional connectivity was carried out using EMDPL. Additional inter-group/condition differences were sought by calculating amplitudes, and ERPs were determined, to verify the presence of neural correlates of true and false memory expected based on the ERP memory literature. The results suggest that the neural mechanisms employed both in the true and in the false memory processing differed between typical participants and those with MCI, with phase synchrony calculated using EMDPL providing the most information about how neural processing differs between groups and conditions. Both behavioural and neurophysiological findings are consistent with the hypothesis that the MCI group relied more on familiarity than the typical participants, for whom evidence pointed to the use of episodic retrieval mechanisms. Furthermore, the findings are consistent with the theory that those with MCI employed compensatory processing to achieve hits. The PL detected during FA suggests that additional search mechanisms were implemented in the absence of identification of a memory trace, and that this took place more readily in the MCI group.

### 1. Behavioural Findings

On DRM free recall, typical participants correctly recalled significantly more presented words than those with MCI, while the latter gave the critical lure significantly more often than the typical group, as found previously [78], [79], [115]. While the same pattern was seen here in the recognition phase, it was not statistically significant. Consistent with our findings, similar recognition levels have been found elsewhere in a DRM task in MCI and controls, and it was postulated that an inter-group difference might have been identified if a free recall component had been included, since recognition requires less cognitive processing than free recall [152]. It is likely here that while episodic memory was required for free recall, after the delay involved in completing 8 word lists, episodic memory was reduced, with gist contributing to recognition. In the free recall stage, greater requirement for episodic memory would have disadvantaged those with MCI compared with typical participants, but the difference in performance on the recognition test may have been reduced, due to relatively less familiarity than episodic memory impairment in MCI [153], [154]. Indeed, while familiarity memory impairment is also recognised in AD, dependence on this type of memory seems to be greater [80], [81], [153], [154]. The results therefore point to the employment of differing types of memory processing in the two groups during the recognition task. There is existing evidence that familiarity (involving gist memory) and recollection (important in free recall) actually have separate neural mechanisms [91], [155], and our results support the notion that these are differentially affected in MCI.

A number of theories have been proposed to account for an increased dependence on gist in MCI. It has been postulated that this results from proportionally greater item-specific memory deterioration [154]. This suggestion is consistent with fuzzy-trace theory, which proposes that episodic memory comprises detail and verbatim information and goes on to predict that gist memory is more persistent than verbatim recollection [153], [154]. According to this theory, a gist representation is constructed during encoding [152]. False recognition would then result from use of the gist information. There is indeed evidence that gist information can be applied to circumvent reduced verbatim, episodic memory [152]. Firstly, the likelihood of false recognition is increased with repeated study lists in those with AD [93]. With repetition of the study list, participants with AD are thought increasingly to build up a semantic gist unchecked by episodic memory of the actual words on the list, while controls are thought to increase their specific, episodic recollections. The expected reduction in semantic memory in AD was accounted for by needing several trials for those with AD to build this gist, which was then used instead of reliable episodic memory [93]. Secondly, false recognition increases when true recognition performance is matched between those with AD and controls [78]. Another theory is that increased false recognition in AD after deep encoding may be due to an inability to use actual recollection to overcome familiarity [80]. Other mechanisms employed in MCI to circumvent reduced episodic memory, that could lead to increases in false recognition include reliance on the plausibility of information [156], and the encoding of generic information [157]. Further evidence for the use of gist was found in false recollection of a picture of an object after presentation of a number of pictures of different versions of the same object [158]. It has been suggested that familiarity results from gist, and that in a typical participant, frontal lobe verification-inhibition mechanisms are used, which are impaired in AD due to frontal lobe dysfunction, leading to greater false recognition [84]. The present study provides both behavioural and neurophysiological evidence in support of an increase in gist memory use in MCI.

Reaction times (RTs) for both hits and FA, were significantly slower in the typical group than the MCI group. A quicker response in the MCI group might suggest an increased dependence on gist memory, as familiarity is usually faster than recollection [159], [160], due to impairment of recollection processes. A model has indeed been proposed, in which a fast response based on familiarity is made, and if there is ambiguity, an extended memory search then ensues [161]. Furthermore, in the typical group, significantly more hits were rated as certain than in the MCI group, which provides further evidence that the hits were achieved through episodic recollection in the typical group as opposed to a heavier reliance on familiarity in the MCI group [91]. While it should be noted that remember/know judgements are not based solely on confidence, words that are remembered with certainty are more likely to reflect remembering than knowing [162]. Therefore many, but not all ‘certain’ words should be remembered. Finally, in the current study, RTs in both groups were found to be longer for hits than for FA. The slower RTs support the use of item-specific recollection in hits, with familiarity or gist playing a greater role in FA.

### 2. Neurophysiological Findings

The neurophysiological findings further support the suggestion that differing neural mechanisms are employed by those with MCI compared with typical controls in true and false memory processing. The right frontal and left parietal regions were selected for region-of-interest analysis, and EMDPL was applied to distinguish episodes of PL in particular frequencies over time. Arbitrary bandpass filter cut-off selection was avoided through adaptive signal decomposition using EMD, which is important, as filter cut-offs have been shown to impact upon whether and when PL is detected [9], [10], as also illustrated here. As predicted, theta and alpha oscillations differed between groups and conditions. Amplitude differences between groups and conditions were not statistically significant, while ERP findings were consistent with expectations based on the literature.

#### 2.1 Phase synchrony

Comparison of PL time-frequency distributions between groups and conditions using the 2D K-S test revealed significant differences in the time-frequency pattern of PL in frontal electrodes between hits and FA in both groups and between the typical and MCI groups for hits. While the neural correlates of true and false memory have been shown to differ in previous studies [34], [68], [107], [108], [110], the present study provides new insights into how they differ and also reveals a difference in processing between adults with MCI and age matched controls. Significantly greater frontal lower theta PL was identified at 475 ms in hits compared with FA in the typical group. We interpret this PL as reflecting the item-specific recollection that is thought to be absent in FA. The timing coincided with that in which left parietal ERPs are thought to reflect item-specific recollection [68], [69]. A parietal ERP component was also identified in the data presented here, peaking at around 320 ms in typical participants during hits. The increased frontal theta PL followed a putative parietal reactivation of a memory trace, and in line with the previous suggested models, this coincided with a greater correlation between frontal and parietal theta and alpha PL levels in this study. It has already been suggested that ERP-components related to short-term memory might result from phase locked theta activity [54]. Our data suggest that frontal theta PL at the time of the previously established parietal ERP component related to item-specific recollection provides a potential mechanism for behavioural memory changes in adults with MCI. Theta oscillations have been identified in the hippocampus during memory processes [163], [164], and hippocampal damaged is well-recognised in AD, thus the changes in theta oscillations demonstrated in our findings are compatible with previous studies.

Three findings here therefore suggest the use of gist memory in MCI may at least in part account for differing neural correlates of memory processing in this group. Firstly, there was more false memory in the MCI group, and the use of gist memory is indeed thought to increase the probability of false memory [160]. Secondly, RTs in the MCI group were on average shorter than in the typical group. Gist is based on familiarity, which is processed earlier than recollection [161]. Thirdly, a greater theta PL in FA than in hits in MCI takes place earlier, at 230 ms, than the theta PL proposed to relate to episodic memory use in hits in the typical group.

The neural processing associated with attention might provide an additional explanation for the behavioural findings. Attention deficits in MCI might lead to reduced ability to inhibit other pathways in the presence of intact semantic networks [78]. Absence of inhibition of a novel (unpresented), yet semantically-related item might lead to its false recall. Deficits in inhibition in MCI and in AD have been identified using the Stroop test [165], [166]. We suggest therefore that frontal theta PL might be a neural correlate of the verification-inhibition mechanism which is impaired in AD, and that this impairment might begin early in the disease process when the person has MCI.

Both semantic memory network breakdown and reduced attentional control and inhibition are consistent with the findings of reduced frontal lobe function in AD, and with the suggestion that impairment in these processes might already be present in participants with MCI, some of whom go on to develop AD. Increased false recognition has indeed been found with frontal lobe damage [81], and increased tendency to exhibit false memory in older compared with younger adults has been suggested to result from frontal lobe dysfunction [77].

Although typical participants produced more hits than adults with MCI in the DRM recognition task, the difference was not statistically significant. We suggest that this might be evidence for the use of compensatory processing in adults with MCI. Increased functional connectivity found in MCI has previously been suggested to be the result of compensatory activity [29], [31]. In addition, significantly higher EEG synchronisation has been shown in the alpha and beta frequency ranges in participants with MCI, which were attributed to compensational mechanisms in adults with MCI, which are no longer effective once AD develops [32]. There were two episodes of increases in theta PL during FA compared to hits in the MCI group, at 230 ms and at 700 ms. This greater PL could be interpreted as reflecting increased searching when a true memory was not activated, and, since this was only present in the group with MCI, it suggests more ready use of compensatory activity when episodic memory is impaired.

At 475 ms, we found both significantly more theta PL during hits than FA, and significantly greater upper alpha PL in FA than in hits in the typical group. Upper alpha oscillations are thought to reflect semantic information processing and long-term memory [55]. They may also play a role in working memory, reflecting transient reactivation of long-term memory [123]. We suggest that when a recent episodic memory is not identified, long-term memory stores including gist information may be accessed, facilitating the occurrence of a false memory. This notion is supported by the significant upper alpha fronto-parietal correlation of PL levels found in hits but not in FA in both groups, which could reflect frontal accessing of a parietally stored long-term memory. The episode of correlation was brief in those with MCI, and we suggest this could be due to accessing of gist rather than a true memory trace, or impairment of this process in the MCI group.

Significantly more parietal PL was found in typical participants than in those with MCI at 350 ms. As already stated, parietal PL might indicate activation of memory storage networks [94]. A greater left parietal activation has also been found elsewhere in true compared with false recognition [68], [110], [167]. Its absence in MCI provides further support for the hypothesis that the neural processing underlying successful hits differs between typical adults and those with MCI.

Anatomical studies have demonstrated that frontal and posterior association cortices are highly interconnected [168]. It has been suggested that prefrontal regions play a role in integrating inputs from various modalities during encoding and at retrieval [169], while the parietal region is thought to be the location where a memory is stored. Fronto-parietal interaction is thought to mediate central executive processes [95], [96], involving attention and long-term memory [21], [59]. One interpretation of our findings, therefore would be that coupling between these areas reflects interaction between storage and central executive processes [20]. When a hit takes place, a successful verification, represented by connectivity, would confirm that the information is old, while in a false memory, this verification does not occur. The increased frontal PL found here in FA compared with hits at around 750 ms in both groups may reflect on-going search for a memory trace when the memory is not available. Failure to find a memory trace would therefore negate the need for connectivity between the parietal and frontal regions, thus providing an explanation for the failure to find significant fronto-parietal correlation in PL during FAs in this study.

In support of this explanation, correlation between frontal and parietal PL levels was significant in the theta frequency range only in the typical group during hits, in which item-specific recollection was achieved. The timing of the correlated activity coincided with a significantly greater frontal theta PL in the typical group during hits compared with FA and followed the greater parietal theta PL in the typical group compared with the MCI group during hits. This might also suggest that the use of gist memory does not necessarily require access to a specific parietally stored memory trace.

Significant correlation of frontal and parietal PL levels also took place in the lower alpha frequency range in hits in both groups. Lower alpha activity has been linked to attention [170], which will be required for hits in this task. It is plausible that reduced attention contributes to FA in both groups. Attention is known to be impaired in AD, and we suggest that the hits in the MCI group either take place despite reduced attention, as attention impairment is only partial, or that attention is less essential when a hit takes place due to the use of gist memory.

Calculation of phase synchrony following two alternative time-frequency decompositions gave rise to two key findings. Firstly, the synchrony found using EMDPL was indeed also found when traditional bandpass filters or wavelets were used, validating the findings using EMDPL. Secondly, EMDPL offered advantages over the other approaches. The precise frequency cut-offs of the filters used did affect whether and when the synchrony was actually detected, and while the timing of phase synchrony detected following wavelet decomposition was similar, time localisation was reduced, as expected using a method in which there is a trade-off between time and frequency localisation. Indeed, a further recent example of better time localisation following EMD than wavelet decomposition is localisation of transcranial magnetic stimulation-evoked EEG oscillations [171]. We concluded that band pass filter- and wavelet-based calculation of PL did not provide any additional information to separate the neural processing between groups and conditions.

#### 2.2 Amplitude changes

We calculated amplitudes using EMD and wavelet time-frequency decomposition. No significant amplitude differences were detected using either method, suggesting that PL provides a more useful approach to assessing the differences in neural processing underlying true and false memory in the two groups. This is consistent with the findings of [21] who demonstrated that increased fronto-parietal theta synchrony found during retrieval in a working memory task did not coincide with a power change. They concluded that the coherence measurement reflected temporal information only.

#### 2.3 ERPs

ERP amplitude was calculated in order to identify the expected P300 component and verify that the data conformed to the literature. Left parietal ERP differences are thought to reflect item-specific recollection [68], [69], and it has been suggested that the greater amplitude in true memory reflects stronger imagery for true memories [107], [108], [110]. Thus, the significantly greater positivity during hits than FA found in the typical group could be interpreted as indicative of retrieval of a true memory.

The same component was also found to have significantly greater amplitude in hits in the typical group than in the MCI group, and the P300 latency in hits in the MCI group was significantly longer than in the typical group. These effects replicate the findings of [109], [111]. These results would also support impairment of reactivation of a true memory in the MCI group.

### 3. Conclusions

The neural correlates of true and false memory processing in participants with mild cognitive impairment were compared with those of age-matched controls. The approach involved EMDPL, which allowed study of the oscillations relating to memory processing without the need for arbitrary bandpass filter cut-off selection. By studying phase information separately from amplitude information, it was possible to study functional connectivity independently from any power changes. Indeed, while no significant inter-group/condition amplitude differences were found, differences in the neural correlates of true and false memory were identified on measurement of PL, and PL differences were also found between typical memory processing and processing by those with MCI.

There was more frontal PL in FA than hits in both groups, and in the MCI group than in the typical group in FA. We have suggested that the former indicates increased searching processes in the absence of retrieval of a true memory and that the latter resulted from differing neural mechanisms underlying retrieval in the MCI group, with an increase in compensatory processing and a greater tendency to rely on gist memory when episodic memory is impaired. Lower correlation between frontal and parietal phase locking levels in the MCI group fits with the notion of AD as a ‘disconnection syndrome’ [2], [25], [28], [58]. That synchrony levels differed within the frontal cortex is in agreement with previous findings of impairment in this region in AD.

Currently there are few tools to determine which patients with MCI will progress to AD. Better prediction of progression to AD is essential for planning patient care, and as advances are made in treatment, for starting preventative measures or symptom-delaying medications before irreversible damage occurs [2]. We suggest that the differing PL levels between groups might be detectable before any other indication of MCI or early AD is present, as the comparable behavioural results between the groups in the DRM recognition task suggest that employment of differing neural processing between the groups compensates for episodic memory decline. We propose that future study could investigate the use of this approach as a screening tool, which could be applied to those deemed, for instance, through a family history of AD, to be at increased risk of developing AD in the future. Long-term follow-up would be required to ascertain whether this method can be used to differentiate between adults with MCI who develop AD and those that do not. Furthermore, the approach could be used to identify those who would benefit from specific treatment approaches. Finally, a greater understanding of the electrophysiological correlates of memory processing should in future allow development of new treatment approaches, including both pharmacological and electrical interventions.

## Supporting Information

Table S1 **Word lists used for the Deese-Roediger-McDermott paradigm.**
(DOC)

Appendix S1 **Topography of ERPs and phase alignment.**
(DOC)
