# Supplementary material for: Neural Correlates of True and False Memory in Mild Cognitive Impairment
Source: PLoS One. 2012 Oct 31;7(10):e48357. doi: 10.1371/journal.pone.0048357 (PMC3485202; doi:10.1371/journal.pone.0048357)
Supplement: Table S1 — Word lists used for the Deese-Roediger-McDermott paradigm. (DOC) [file pone.0048357.s001.doc]

**Supporting Information**

Table S1.

**Lure: window**

| **Presented** | **Recognition** | |
| --- | --- | --- |
|  | **New** | **Old** |
| door | window | door |
| glass | shutter | glass |
| pane | sill | pane |
| ledge | shade | ledge |
| house | frame | curtain |
| open | breeze | view |
| curtain |  |  |
| view |  |  |
| sash |  |  |
| screen |  |  |

Table S2.

**Lure:** soft

| **Presented** | **Recognition** | |
| --- | --- | --- |
|  | **New** | **Old** |
| hard | soft | hard |
| pillow | cotton | pillow |
| silky | skin | loud |
| loud | kitten | touch |
| light | furry | downy |
| fur | feather | fluffy |
| touch |  |  |
| fluffy |  |  |
| downy |  |  |
| tender |  |  |

Table S3.

**Lure: anger**

| **Presented** | **Recognition** | |
| --- | --- | --- |
|  | **New** | **Old** |
| rage | anger | rage |
| temper | enrage | temper |
| fury | fight | fury |
| ire | mad | happy |
| wrath | fear | calm |
| happy | hate | emotion |
| hatred |  |  |
| mean |  |  |
| calm |  |  |
| emotion |  |  |

Table S4.

**Lure: pen** (reversed list)

| **Presented** | **Recognition** | |
| --- | --- | --- |
|  | **New** | **Old** |
| pen | pencil | pen |
| quill | write | quill |
| biro | fountain | biro |
| red | shade | red |
| letter | felt | letter |
| cap | scribble | cap |
| crayon |  |  |
| refill |  |  |
| tip |  |  |
| marker |  |  |

Table S5.

Lure: doctor

| **Presented** | **Recognition** | |
| --- | --- | --- |
|  | **New** | **Old** |
| nurse | doctor | nurse |
| sick | surgeon | sick |
| lawyer | clinic | lawyer |
| medicine | ill | medicine |
| health | office | dentist |
| hospital | cure | physician |
| dentist |  |  |
| physician |  |  |
| patient |  |  |

Table S6.

**Lure:** needle

| **Presented** | **Recognition** | |
| --- | --- | --- |
|  | **New** | **Old** |
| thread | needle | thread |
| pin | prick | pin |
| eye | thimble | haystack |
| sewing | point | injection |
| sharp | thorn | syringe |
| haystack | hurt | knitting |
| injection |  |  |
| syringe |  |  |
| cloth |  |  |
| knitting |  |  |

Table S7.

**Lure:** chair

| **Presented** | **Recognition** | |
| --- | --- | --- |
|  | **New** | **Old** |
| table | chair | table |
| sit | stool | sit |
| seat | bench | seat |
| couch | legs | couch |
| desk | wood | desk |
| furniture | rocking | sofa |
| sofa |  |  |
| cushion |  |  |
| swivel |  |  |
| sitting |  |  |

Table S8.

**Lure: man** (reversed list)

| **Presented** | **Recognition** | |
| --- | --- | --- |
|  | **New** | **Old** |
| man | woman | man |
| person | uncle | person |
| handsome | lady | handsome |
| strong | mouse | strong |
| friend | male | friend |
| husband | father | husband |
| beard |  |  |
| muscle |  |  |
| suit |  |  |
| old |  |  |

Table S9.

**Lure:** smell

| **Presented** | **Recognition** | |
| --- | --- | --- |
|  | **New** | **Old** |
| nose | smell | nose |
| sniff | fragrance | sniff |
| aroma | perfume | aroma |
| scent | rose | scent |
| stench | breathe | stench |
| whiff | salts | whiff |
| hear |  |  |
| see |  |  |
| nostril |  |  |
| reek |  |  |

Table S10.

**Lure: river** (reversed list)

| **Presented** | **Recognition** | |
| --- | --- | --- |
|  | **New** | **Old** |
| river | water | river |
| bridge | stream | bridge |
| brook | lake | brook |
| fish | Thames | fish |
| winding | boat | winding |
| barge | ride | barge |
| swim |  |  |
| flow |  |  |
| run |  |  |
| creek |  |  |

Table S11.

**Lure:** sleep

| **Presented** | **Recognition** | |
| --- | --- | --- |
|  | **New** | **Old** |
| bed | sleep | bed |
| rest | drowsy | rest |
| tired | nap | tired |
| dream | peace | dream |
| wake | awake | wake |
| snooze | blanket | snooze |
| doze |  |  |
| slumber |  |  |
| snore |  |  |
| yawn |  |  |

Table S12.

Lure: smoke

| **Presented** | **Recognition** | |
| --- | --- | --- |
|  | **New** | **Old** |
| cigarette | smoke | cigarette |
| puff | pipe | puff |
| blaze | stink | cigar |
| billows | lungs | chimney |
| pollution | flames | tobacco |
| ashes | stain | ashes |
| cigar |  |  |
| chimney |  |  |
| fire |  |  |
| tobacco |  |  |

Table S13.

**Lure:** spider

| **Presented** | **Recognition** | |
| --- | --- | --- |
|  | **New** | **Old** |
| spider | web | spider |
| cobweb | insect | cobweb |
| tarantula | bug | tarantula |
| beetle | fright | beetle |
| crawly | fly | crawly |
| small | arachnid | small |
| poison |  |  |
| bite |  |  |
| creepy |  |  |
| animal |  |  |

Table S14.

Lure: slow

| **Presented** | **Recognition** | |
| --- | --- | --- |
|  | **New** | **Old** |
| fast | slow | fast |
| lethargic | snail | lethargic |
| sluggish | quick | sluggish |
| listless | cautious | listless |
| delay | stop | delay |
| dawdle | wait | dawdle |
| tortoise |  |  |
| hesitant |  |  |
| speed |  |  |
| traffic |  |  |

Table S15.

**Lure:** cup

| **Presented** | **Recognition** | |
| --- | --- | --- |
|  | **New** | **Old** |
| mug | cup | mug |
| saucer | coffee | saucer |
| tea | plastic | tea |
| measuring | handle | measuring |
| coaster | straw | coaster |
| lid | drink | lid |
| goblet |  |  |
| soup |  |  |
| beaker |  |  |

Table S16.

Lure: thief

| **Presented** | **Recognition** | |
| --- | --- | --- |
|  | **New** | **Old** |
| steal | thief | steal |
| robber | bandit | robber |
| crook | bank | crook |
| burglar | crime | burglar |
| money | villain | criminal |
| cop | gun | cop |
| bad |  |  |
| rob |  |  |
| jail |  |  |
| criminal |  |  |

Table S17.

**Lure:** cold

| **Presented** | **Recognition** | |
| --- | --- | --- |
|  | **New** | **Old** |
| hot | cold | hot |
| snow | Arctic | snow |
| warm | frost | warm |
| winter | weather | winter |
| ice | chilly | ice |
| wet | air | wet |
| frigid |  |  |
| heat |  |  |
| freeze |  |  |
| shiver |  |  |

Table S18.

Lure: rough

| **Presented** | **Recognition** | |
| --- | --- | --- |
|  | **New** | **Old** |
| smooth | rough | smooth |
| bumpy | sandpaper | bumpy |
| road | uneven | harsh |
| tough | gravel | coarse |
| jagged | choppy | jagged |
| harsh | ground | abrasive |
| coarse |  |  |
| abrasive |  |  |
| rugged |  |  |
| terrain |  |  |

Table S19.

**Lure: bread** (reversed list)

| **Presented** | **Recognition** | |
| --- | --- | --- |
|  | **New** | **Old** |
| bread | butter | bread |
| loaf | sandwich | loaf |
| toast | rye | toast |
| food | jam | food |
| eat | milk | eat |
| wine | flour | wine |
| marmalade |  |  |
| dough |  |  |
| crust |  |  |
| slice |  |  |

Table S20.

Lure: high

| **Presented** | **Recognition** | |
| --- | --- | --- |
|  | **New** | **Old** |
| low | high | low |
| clouds | jump | clouds |
| noon | tower | noon |
| tall | dive | tall |
| building | sky | building |
| airplane | cliff | jump |
| above |  |  |
| elevate |  |  |
| up |  |  |
| over |  |  |

Table S21.

**Lure:** mountain

| **Presented** | **Recognition** | |
| --- | --- | --- |
|  | **New** | **Old** |
| hill | mountain | hill |
| valley | climber | valley |
| climb | peak | climb |
| summit | glacier | summit |
| top | goat | top |
| molehill | range | molehill |
| plain |  |  |
| bike |  |  |
| steep |  |  |
| ski |  |  |

Table S22.

**Lure: foot** (reversed list)

| **Presented** | **Recognition** | |
| --- | --- | --- |
|  | **New** | **Old** |
| foot | shoe | foot |
| inch | hand | inch |
| sock | toe | sock |
| smell | kick | smell |
| mouth | sandals | mouth |
| arm | soccer | arm |
| yard |  |  |
| walk |  |  |
| ankle |  |  |
| boot |  |  |

Table S23.

**Lure: sweet**

| **Presented** | **Recognition** | |
| --- | --- | --- |
|  | **New** | **Old** |
| sour | sweet | sour |
| candy | tart | candy |
| sugar | chocolate | sugar |
| bitter | heart | bitter |
| good | cake | honey |
| taste | pie | taste |
| tooth |  |  |
| nice |  |  |
| honey |  |  |
| fudge |  |  |

Table S24.

Lure: music

| **Presented** | **Recognition** | |
| --- | --- | --- |
|  | **New** | **Old** |
| note | music | note |
| sound | rhythm | sound |
| piano | orchestra | piano |
| radio | art | radio |
| band | horn | band |
| melody | sing | melody |
| concert |  |  |
| instrument |  |  |
| symphony |  |  |
| jazz |  |  |
