# Supplementary material for: Neural Correlates of True and False Memory in Mild Cognitive Impairment
Source: PLoS One. 2012 Oct 31;7(10):e48357. doi: 10.1371/journal.pone.0048357 (PMC3485202; doi:10.1371/journal.pone.0048357)
Supplement: Appendix S1 — Topography of ERPs and phase alignment. (DOC) [file pone.0048357.s002.doc]

Appendix S1.

In order to verify task-dependent activity recorded by the 8 electrodes chosen on the basis of the literature for this study, ERPs and phase alignment were calculated for all 128 sensors for each typical participant for hits and for FA. The data were first pre-processed as described in Section 2.6.

ERPs for each electrode were calculated as detailed in Section 2.11, for 250ms time windows with 50ms overlap. A T-test as described in Section 2.8.2 was then applied, and p-values were plotted for each electrode (Fig. 2). ERPs differed between hits and FA in electrodes 119 and 124 in the right frontal area and electrode 60 in the left parietal area 0-250ms post-stimulus (Fig. 2A) and in electrodes 124 in the right frontal area and electrodes 60 and 67 in the left parietal area (Fig. 2B).

To calculate phase alignment, the pre-processed data were filtered using a traditional Butterworth bandpass filter to identify theta (4-8 Hz) and alpha (8-12 Hz) frequency bands. The Hilbert transform was then applied, as described in Section 2.7, to yield a phase series for each trial. Phase alignment was then calculated for each time point as the length of the unit phase vector across trials for each participant, divided by the trial number, returning an index of phase alignment from 0 indicating no phase alignment, to 1 indicating complete phase alignment [18]. Mean phase alignments over participants for each condition over 250ms windows with 50ms overlap were calculated, and a two-sample T-test was again applied to compare phase alignment in hits versus FA. A difference in theta phase alignment was seen in the first 250ms post-stimulus in electrodes 3 and 4 in the right frontal area and in electrode 59 in the left parietal area (Fig. 2C). An alpha phase alignment difference was seen from 150-400ms post-stimulus in electrodes 60, 66, and 67 in the left parietal area (Fig. 2D).

It should be emphasised that electrode choice was based on the literature. The above does not constitute an extensive spatial analysis but rather an indication that the electrodes selected did indeed record activity that differed between conditions.
